# 10,21-Dimethyl-2,7,13,18-tetraphenyl-3,6,14,17-tetraazatricyclo[17.3.1.1^8,12^]tetracosa-1(23),2,6,8(24),9,11,13,17,19,21-decaene-23,24-diol cyclohexane 0.33-solvate

**DOI:** 10.1107/S1600536811036622

**Published:** 2011-09-30

**Authors:** Ashish K. Asatkar, Vinay K. Verma, Tripti A. Jain, Rajendra Singh, Sushil K. Gupta, Ray J. Butcher

**Affiliations:** a Department of Chemistry, Dish Institute of Management and Technology, Raipur 492101(C.G.), India; b Directorate of ER & IPR, Defence Research and Development Organisation, New Delhi 110105, India; c School of Studies in Chemistry, Jiwaji University, Gwalior 474011, India; d Department of Chemistry, Howard University, Washington DC 20059, USA

## Abstract

The title compound, C_46_H_40_N_4_O_2_·0.33C_6_H_12_, was obtained unintentionally as a product of an attempted synthesis of a cadmium(II) complex of the [2,6-{PhSe(CH_2_)_2_N=CPh}_2_C_6_H_2_(4-Me)(OH)] ligand. The full tetra­imino­diphenol macrocyclic ligand is generated by the application of an inversion centre. The macrocyclic ligand features strong intra­molecular O—H⋯N hydrogen bonds. The dihedral angles formed between the phenyl ring incorporated within the macrocycle and the peripheral phenyl rings are 82.99 (8) and 88.20 (8)°. The cyclo­hexane solvent mol­ecule lies about a site of 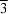 symmetry. Other solvent within the lattice was disordered and was treated with the SQUEEZE routine [Spek (2009). *Acta Cryst.* D**65**, 148–155].

## Related literature

For information on phenol-based Schiff base ligands, complexes and their applications, see: Vigato *et al.* (2007 ▶); Fenton *et al.* (2010 ▶); Avaji *et al.* (2009 ▶); Na *et al.* (2006 ▶); Dutta *et al.* (2004 ▶); Mandal *et al.* (1989 ▶); Gupta *et al.* (2002 ▶, 2010 ▶).
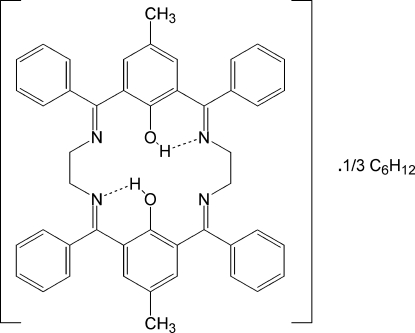


## Experimental

### 

#### Crystal data

3C_46_H_40_N_4_O_2_·C_6_H_12_
*M* *_r_* = 2126.62
Rhombohedral, 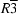

*a* = 28.0966 (2) Å
*c* = 16.0265 (2) Å
α = 90°
γ = 120°
*V* = 10956.6 (2) Å^3^
*Z* = 3
Mo *K*α radiation
μ = 0.06 mm^−1^
*T* = 295 K
0.44 × 0.41 × 0.32 mm

#### Data collection

Oxford Diffraction Gemini R diffractometer
Absorption correction: multi-scan (*CrysAlis RED*; Oxford Diffraction, 2009 ▶) *T*_min_ = 0.173, *T*_max_ = 1.000
10270 measured reflections
5016 independent reflections
3022 reflections with *I* > 2σ(*I*)
*R*_int_ = 0.020

#### Refinement

*R*[*F* ^2^ > 2σ(*F* ^2^)] = 0.063
*wR*(*F* ^2^) = 0.222
*S* = 0.93
5016 reflections
246 parameters
H-atom parameters constrained
Δρ_max_ = 0.85 e Å^−3^
Δρ_min_ = −0.27 e Å^−3^

### 

Data collection: *CrysAlis CCD* (Oxford Diffraction, 2009 ▶); cell refinement: *CrysAlis RED* (Oxford Diffraction, 2009 ▶); data reduction: *CrysAlis RED*; program(s) used to solve structure: *SHELXS97* (Sheldrick, 2008 ▶); program(s) used to refine structure: *SHELXL97* (Sheldrick, 2008 ▶) and *PLATON* (Spek, 2009 ▶); molecular graphics: *SHELXTL* (Sheldrick, 2008 ▶); software used to prepare material for publication: *SHELXTL*.

## Supplementary Material

Crystal structure: contains datablock(s) I, global. DOI: 10.1107/S1600536811036622/tk2788sup1.cif

Structure factors: contains datablock(s) I. DOI: 10.1107/S1600536811036622/tk2788Isup2.hkl

Supplementary material file. DOI: 10.1107/S1600536811036622/tk2788Isup3.cml

Additional supplementary materials:  crystallographic information; 3D view; checkCIF report

## Figures and Tables

**Table 1:** Hydrogen-bond geometry (Å, °)

*D*—H⋯*A*	*D*—H	H⋯*A*	*D*⋯*A*	*D*—H⋯*A*
O1—H1*O*⋯N1*A*	0.82	1.81	2.532 (2)	146

**Fig. 1.**
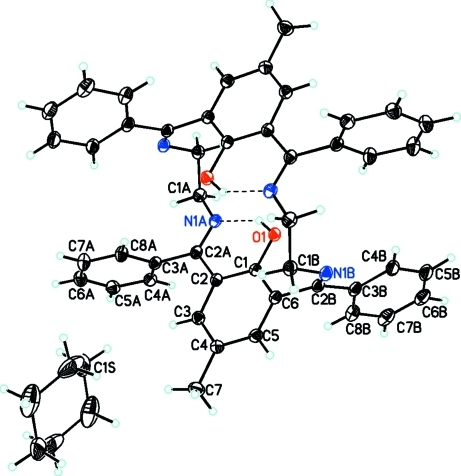
The molecular structure of the title compound with atom numbering scheme. Displacement ellipsoids are drawn at 30% probability level. The cyclohexane ring has 3 symmetry.

## supplementary crystallographic information

### Comment

Schiff bases have played an important role in the development of coordination chemistry related to catalysis and enzymatic reactions, magnetism and supramolecular architectures (Vigato *et al.*, 2007; Fenton *et al.*, 2010; Avaji *et al.*, 2009; Na *et al.*, 2006; Dutta *et al.*, 2004; Mandal *et al.*, 1989). Very recently we have reported a dinuclear copper (II) complex with a neutral tetraiminodiphenol macrocycle with a C2 lateral chain (Gupta *et al.*, 2010). We herein report the synthesis and crystal structure of its Schiff base ligand, Fig. 1. The phenolic hydrogen forms an intramolecular hydrogen bond with N1 of the imino group, Table 1. The C1—O1 bond [1.342 (2) Å] appears to be shorter than the equivalent bond in the related structure, (PhCO)_2_C_6_H_2_(OH)(4-Me) [1.360 (4) Å] (Gupta *et al.*, 2002). The imino groups are coplanar with the phenyl ring to which they are attached. The dihedral angles between the phenyl moiety which is part of the macrocycle and the peripheral phenyl rings are 82.99 (8) and 88.20 (8) °. The crystals contain cyclohexane solvent molecules which lie on a site of 3 symmetry and thus only one atom is unique and a chair conformation is imposed.

### Experimental

A methanolic solution of ethylenediamine (0.13 ml, 1.94 mmol) was added drop wise to a suspension of CdCl_2_.H_2_O (0.194 g, 0.97 mmol) in methanol and stirred for 5 min. A methanolic solution of [2,6-{PhSe(CH_2_)_2_N═ CPh}_2_C_6_H_2_(4-Me)(OH)] (0.66 g, 0.97 mmol) was added drop wise to the above reaction mixture with constant stirring under Ar atmosphere. The reaction was carried out at room temperature while stirring vigorously. After stirring the reaction mixture for 12 h, the precipitate thus obtained was filtered, dried under vacuum and isolated as the mixture of solid compounds. The macrocycle from the mixture was separated by dissolving it in warm chloroform. Crystals suitable were grown by slow evaporation of its solution in chloroform-cyclohexane mixture (9:1 *v*/*v*), giving rise to yellow regular cubic crystals in 45% yield. Anal. Calcd. for C_48_H_44_N_4_O_2_: C, 81.30; H, 6.21; N, 7.90%. Found: C, 81.61; H, 6.41; N, 8.02.

### Refinement

C-bound H atoms were located at their idealized positions and were included in the final structural model in riding-motion approximation with d(C—H) = 0.93 Å for aromatic CH, 0.97 Å for CH_2_ groups, 0.96 Å for CH_3_ groups, and d(O—H) = 0.82 Å, and with *U*_iso_(H) = 1.2U_eq_(C) or *U*_iso_(H) = 1.5*U*_eq_(methyl-C and O). The structure contained disordered solvent molecules located near symmetry elements. These were not able to be resolved and so were removed using the SQUEEZE routine in *PLATON* (Spek, 2009).

### Crystal data

**Table:**

3C_46_H_40_N_4_O_2_·C_6_H_12_	*F*(000) = 3384
*M**_r_* = 2126.62	*D*_x_ = 0.967 Mg m^−^^3^
Rhombohedral, *R*3	Mo *K*α radiation, λ = 0.71073 Å
Hall symbol: -R 3	Cell parameters from 3689 reflections
*a* = 28.0966 (2) Å	θ = 4.2–77.4°
*c* = 16.0265 (2) Å	µ = 0.06 mm^−^^1^
α = 90°	*T* = 295 K
γ = 120°	Block, yellow
*V* = 10956.6 (2) Å^3^	0.44 × 0.41 × 0.32 mm
*Z* = 3	

### Data collection

**Table:**

Oxford Diffraction Gemini R diffractometer	5016 independent reflections
Radiation source: fine-focus sealed tube	3022 reflections with *I* > 2σ(*I*)
graphite	*R*_int_ = 0.020
Detector resolution: 10.5081 pixels mm^-1^	θ_max_ = 26.8°, θ_min_ = 2.1°
φ and ω scans	*h* = −34→33
Absorption correction: multi-scan (*CrysAlis RED*; Oxford Diffraction, 2009)	*k* = −35→31
*T*_min_ = 0.173, *T*_max_ = 1.000	*l* = −17→19
10270 measured reflections	

### Refinement

**Table:**

Refinement on *F*^2^	Primary atom site location: structure-invariant direct methods
Least-squares matrix: full	Secondary atom site location: difference Fourier map
*R*[*F*^2^ > 2σ(*F*^2^)] = 0.063	Hydrogen site location: inferred from neighbouring sites
*wR*(*F*^2^) = 0.222	H-atom parameters constrained
*S* = 0.93	*w* = 1/[σ^2^(*F*_o_^2^) + (0.1584*P*)^2^] where *P* = (*F*_o_^2^ + 2*F*_c_^2^)/3
5016 reflections	(Δ/σ)_max_ < 0.001
246 parameters	Δρ_max_ = 0.85 e Å^−^^3^
0 restraints	Δρ_min_ = −0.27 e Å^−^^3^

### Special details

**Table:**

Geometry. All e.s.d.'s (except the e.s.d. in the dihedral angle between two l.s. planes) are estimated using the full covariance matrix. The cell e.s.d.'s are taken into account individually in the estimation of e.s.d.'s in distances, angles and torsion angles; correlations between e.s.d.'s in cell parameters are only used when they are defined by crystal symmetry. An approximate (isotropic) treatment of cell e.s.d.'s is used for estimating e.s.d.'s involving l.s. planes.
Refinement. Refinement of *F*^2^ against ALL reflections. The weighted *R*-factor *wR* and goodness of fit *S* are based on *F*^2^, conventional *R*-factors *R* are based on *F*, with *F* set to zero for negative *F*^2^. The threshold expression of *F*^2^ > σ(*F*^2^) is used only for calculating *R*-factors(gt) *etc*. and is not relevant to the choice of reflections for refinement. *R*-factors based on *F*^2^ are statistically about twice as large as those based on *F*, and *R*- factors based on ALL data will be even larger.

### Fractional atomic coordinates and isotropic or equivalent isotropic displacement parameters (Å^2^)

**Table:**

	*x*	*y*	*z*	*U*_iso_*/*U*_eq_	
O1	0.00757 (6)	0.45286 (6)	0.94763 (8)	0.0730 (4)	
H1O	−0.0035	0.4423	0.9952	0.088*	
N1A	0.01498 (8)	0.43270 (7)	1.09956 (10)	0.0711 (4)	
N1B	0.04471 (7)	0.53952 (7)	0.76712 (10)	0.0743 (5)	
C1	0.06233 (8)	0.48575 (8)	0.94867 (12)	0.0624 (4)	
C2	0.09343 (8)	0.49450 (8)	1.02149 (12)	0.0645 (5)	
C3	0.15063 (9)	0.52866 (9)	1.01686 (13)	0.0724 (5)	
H3A	0.1714	0.5343	1.0648	0.087*	
C4	0.17723 (9)	0.55424 (9)	0.94371 (14)	0.0757 (5)	
C5	0.14523 (9)	0.54553 (9)	0.87394 (13)	0.0752 (5)	
H5A	0.1624	0.5629	0.8244	0.090*	
C6	0.08866 (8)	0.51206 (8)	0.87453 (12)	0.0669 (5)	
C7	0.23917 (11)	0.59108 (13)	0.9403 (2)	0.1046 (9)	
H7A	0.2548	0.5740	0.9068	0.157*	
H7B	0.2539	0.5968	0.9958	0.157*	
H7C	0.2480	0.6258	0.9161	0.157*	
C1A	−0.01466 (10)	0.40063 (10)	1.17263 (14)	0.0781 (6)	
H1AA	0.0100	0.4121	1.2202	0.094*	
H1AB	−0.0275	0.3620	1.1624	0.094*	
C2A	0.06650 (9)	0.46672 (8)	1.10066 (12)	0.0663 (5)	
C3A	0.10126 (9)	0.48103 (9)	1.17789 (12)	0.0694 (5)	
C4A	0.11904 (9)	0.44689 (10)	1.20864 (14)	0.0775 (6)	
H4AA	0.1107	0.4147	1.1806	0.093*	
C5A	0.14966 (11)	0.46100 (12)	1.28222 (16)	0.0909 (7)	
H5AA	0.1621	0.4382	1.3030	0.109*	
C6A	0.16148 (12)	0.50778 (13)	1.32378 (18)	0.0991 (8)	
H6AA	0.1817	0.5168	1.3730	0.119*	
C7A	0.14353 (14)	0.54175 (13)	1.29287 (19)	0.1052 (9)	
H7AA	0.1517	0.5738	1.3214	0.126*	
C8A	0.11359 (12)	0.52882 (11)	1.22012 (15)	0.0878 (7)	
H8AA	0.1017	0.5521	1.1995	0.105*	
C1B	0.06337 (9)	0.59170 (9)	0.80786 (13)	0.0762 (6)	
H1BA	0.0890	0.6212	0.7719	0.091*	
H1BB	0.0824	0.5931	0.8591	0.091*	
C2B	0.05487 (8)	0.50364 (8)	0.79799 (12)	0.0689 (5)	
C3B	0.03317 (10)	0.45037 (9)	0.75241 (13)	0.0770 (6)	
C4B	−0.00983 (11)	0.43386 (12)	0.69686 (16)	0.0958 (8)	
H4BA	−0.0262	0.4553	0.6892	0.115*	
C5B	−0.02877 (14)	0.38494 (14)	0.6522 (2)	0.1131 (10)	
H5BA	−0.0584	0.3736	0.6161	0.136*	
C6B	−0.00516 (16)	0.35455 (13)	0.6604 (2)	0.1179 (12)	
H6BA	−0.0172	0.3230	0.6283	0.141*	
C7B	0.03724 (17)	0.36951 (12)	0.7163 (2)	0.1160 (11)	
H7BA	0.0533	0.3477	0.7229	0.139*	
C8B	0.05599 (13)	0.41755 (11)	0.76307 (17)	0.0957 (8)	
H8BA	0.0841	0.4274	0.8016	0.115*	
C1S	0.2722 (4)	0.6162 (8)	1.1813 (4)	0.244 (6)	
H1SA	0.2686	0.6132	1.2416	0.293*	
H1SB	0.2379	0.5879	1.1573	0.293*	

### Atomic displacement parameters (Å^2^)

**Table:**

	*U*^11^	*U*^22^	*U*^33^	*U*^12^	*U*^13^	*U*^23^
O1	0.0741 (8)	0.0811 (9)	0.0525 (7)	0.0303 (7)	0.0009 (6)	0.0076 (6)
N1A	0.0838 (11)	0.0741 (10)	0.0554 (9)	0.0395 (9)	0.0047 (8)	0.0089 (7)
N1B	0.0852 (11)	0.0795 (10)	0.0536 (9)	0.0378 (9)	0.0032 (8)	0.0041 (7)
C1	0.0697 (11)	0.0624 (9)	0.0551 (10)	0.0330 (9)	0.0014 (8)	0.0006 (7)
C2	0.0766 (11)	0.0649 (10)	0.0563 (10)	0.0387 (9)	0.0002 (8)	0.0008 (8)
C3	0.0747 (12)	0.0779 (12)	0.0679 (12)	0.0407 (10)	−0.0049 (9)	−0.0011 (9)
C4	0.0710 (12)	0.0788 (12)	0.0751 (13)	0.0358 (10)	0.0028 (10)	0.0010 (10)
C5	0.0795 (13)	0.0786 (12)	0.0645 (12)	0.0372 (10)	0.0110 (9)	0.0067 (10)
C6	0.0750 (11)	0.0684 (10)	0.0557 (10)	0.0347 (9)	0.0045 (8)	0.0023 (8)
C7	0.0771 (15)	0.119 (2)	0.1015 (19)	0.0369 (14)	0.0056 (13)	0.0134 (16)
C1A	0.0922 (14)	0.0802 (13)	0.0623 (12)	0.0433 (11)	0.0065 (10)	0.0161 (9)
C2A	0.0833 (13)	0.0694 (11)	0.0554 (10)	0.0452 (10)	−0.0006 (8)	0.0010 (8)
C3A	0.0833 (12)	0.0795 (12)	0.0524 (9)	0.0460 (10)	0.0008 (8)	0.0044 (8)
C4A	0.0883 (14)	0.0843 (13)	0.0722 (13)	0.0523 (12)	−0.0018 (10)	0.0034 (10)
C5A	0.0928 (15)	0.1126 (19)	0.0818 (15)	0.0622 (14)	−0.0079 (12)	0.0121 (14)
C6A	0.1021 (18)	0.120 (2)	0.0752 (15)	0.0557 (17)	−0.0221 (13)	−0.0040 (14)
C7A	0.133 (2)	0.1024 (18)	0.0819 (16)	0.0597 (18)	−0.0228 (16)	−0.0222 (14)
C8A	0.1165 (18)	0.0890 (15)	0.0726 (13)	0.0622 (14)	−0.0160 (13)	−0.0088 (11)
C1B	0.0842 (13)	0.0747 (12)	0.0632 (11)	0.0349 (10)	0.0047 (10)	0.0084 (9)
C2B	0.0748 (11)	0.0757 (11)	0.0480 (9)	0.0315 (9)	0.0094 (8)	0.0048 (8)
C3B	0.0891 (14)	0.0745 (12)	0.0540 (10)	0.0309 (11)	0.0141 (9)	0.0006 (9)
C4B	0.0947 (16)	0.1020 (17)	0.0703 (14)	0.0340 (14)	0.0022 (12)	−0.0152 (12)
C5B	0.107 (2)	0.109 (2)	0.0857 (18)	0.0260 (17)	0.0073 (15)	−0.0288 (16)
C6B	0.124 (2)	0.0865 (18)	0.098 (2)	0.0187 (17)	0.0379 (19)	−0.0227 (15)
C7B	0.156 (3)	0.0860 (17)	0.099 (2)	0.0557 (19)	0.036 (2)	0.0008 (15)
C8B	0.125 (2)	0.0843 (15)	0.0737 (15)	0.0490 (15)	0.0074 (14)	−0.0013 (12)
C1S	0.195 (7)	0.318 (16)	0.099 (4)	0.037 (6)	−0.012 (4)	−0.054 (8)

### Geometric parameters (Å, °)

**Table:**

O1—C1	1.342 (2)	C4A—H4AA	0.9300
O1—H1O	0.8200	C5A—C6A	1.358 (4)
N1A—C2A	1.275 (3)	C5A—H5AA	0.9300
N1A—C1A	1.458 (2)	C6A—C7A	1.375 (4)
N1B—C2B	1.277 (3)	C6A—H6AA	0.9300
N1B—C1B	1.443 (3)	C7A—C8A	1.376 (4)
C1—C6	1.400 (3)	C7A—H7AA	0.9300
C1—C2	1.404 (3)	C8A—H8AA	0.9300
C2—C3	1.402 (3)	C1B—C1A^i^	1.521 (3)
C2—C2A	1.484 (3)	C1B—H1BA	0.9700
C3—C4	1.383 (3)	C1B—H1BB	0.9700
C3—H3A	0.9300	C2B—C3B	1.494 (3)
C4—C5	1.378 (3)	C3B—C8B	1.372 (4)
C4—C7	1.517 (3)	C3B—C4B	1.381 (4)
C5—C6	1.384 (3)	C4B—C5B	1.398 (4)
C5—H5A	0.9300	C4B—H4BA	0.9300
C6—C2B	1.496 (3)	C5B—C6B	1.324 (5)
C7—H7A	0.9600	C5B—H5BA	0.9300
C7—H7B	0.9600	C6B—C7B	1.378 (5)
C7—H7C	0.9600	C6B—H6BA	0.9300
C1A—C1B^i^	1.520 (3)	C7B—C8B	1.396 (4)
C1A—H1AA	0.9700	C7B—H7BA	0.9300
C1A—H1AB	0.9700	C8B—H8BA	0.9300
C2A—C3A	1.502 (3)	C1S—C1S^ii^	1.657 (7)
C3A—C4A	1.375 (3)	C1S—C1S^iii^	1.658 (7)
C3A—C8A	1.384 (3)	C1S—H1SA	0.9700
C4A—C5A	1.395 (3)	C1S—H1SB	0.9700
			
C1—O1—H1O	109.5	C4A—C5A—H5AA	119.8
C2A—N1A—C1A	122.38 (18)	C5A—C6A—C7A	119.9 (2)
C2B—N1B—C1B	121.06 (18)	C5A—C6A—H6AA	120.1
O1—C1—C6	118.32 (17)	C7A—C6A—H6AA	120.1
O1—C1—C2	122.01 (17)	C6A—C7A—C8A	120.7 (3)
C6—C1—C2	119.67 (18)	C6A—C7A—H7AA	119.7
C3—C2—C1	118.41 (18)	C8A—C7A—H7AA	119.7
C3—C2—C2A	120.92 (18)	C7A—C8A—C3A	119.6 (2)
C1—C2—C2A	120.65 (18)	C7A—C8A—H8AA	120.2
C4—C3—C2	122.5 (2)	C3A—C8A—H8AA	120.2
C4—C3—H3A	118.8	N1B—C1B—C1A^i^	109.98 (19)
C2—C3—H3A	118.8	N1B—C1B—H1BA	109.7
C5—C4—C3	117.41 (19)	C1A^i^—C1B—H1BA	109.7
C5—C4—C7	121.1 (2)	N1B—C1B—H1BB	109.7
C3—C4—C7	121.5 (2)	C1A^i^—C1B—H1BB	109.7
C4—C5—C6	122.7 (2)	H1BA—C1B—H1BB	108.2
C4—C5—H5A	118.6	N1B—C2B—C3B	117.44 (19)
C6—C5—H5A	118.6	N1B—C2B—C6	124.58 (19)
C5—C6—C1	119.25 (19)	C3B—C2B—C6	117.94 (19)
C5—C6—C2B	121.62 (18)	C8B—C3B—C4B	118.6 (2)
C1—C6—C2B	119.13 (17)	C8B—C3B—C2B	121.2 (2)
C4—C7—H7A	109.5	C4B—C3B—C2B	120.1 (2)
C4—C7—H7B	109.5	C3B—C4B—C5B	120.0 (3)
H7A—C7—H7B	109.5	C3B—C4B—H4BA	120.0
C4—C7—H7C	109.5	C5B—C4B—H4BA	120.0
H7A—C7—H7C	109.5	C6B—C5B—C4B	121.1 (3)
H7B—C7—H7C	109.5	C6B—C5B—H5BA	119.5
N1A—C1A—C1B^i^	110.73 (17)	C4B—C5B—H5BA	119.5
N1A—C1A—H1AA	109.5	C5B—C6B—C7B	120.2 (3)
C1B^i^—C1A—H1AA	109.5	C5B—C6B—H6BA	119.9
N1A—C1A—H1AB	109.5	C7B—C6B—H6BA	119.9
C1B^i^—C1A—H1AB	109.5	C6B—C7B—C8B	119.6 (3)
H1AA—C1A—H1AB	108.1	C6B—C7B—H7BA	120.2
N1A—C2A—C2	118.15 (18)	C8B—C7B—H7BA	120.2
N1A—C2A—C3A	123.70 (18)	C3B—C8B—C7B	120.4 (3)
C2—C2A—C3A	118.15 (18)	C3B—C8B—H8BA	119.8
C4A—C3A—C8A	119.9 (2)	C7B—C8B—H8BA	119.8
C4A—C3A—C2A	121.5 (2)	C1S^ii^—C1S—C1S^iii^	112.3 (4)
C8A—C3A—C2A	118.47 (18)	C1S^ii^—C1S—H1SA	109.1
C3A—C4A—C5A	119.5 (2)	C1S^iii^—C1S—H1SA	109.1
C3A—C4A—H4AA	120.3	C1S^ii^—C1S—H1SB	109.1
C5A—C4A—H4AA	120.3	C1S^iii^—C1S—H1SB	109.1
C6A—C5A—C4A	120.5 (2)	H1SA—C1S—H1SB	107.9
C6A—C5A—H5AA	119.8		
			
O1—C1—C2—C3	178.78 (18)	C8A—C3A—C4A—C5A	−0.3 (4)
C6—C1—C2—C3	−1.5 (3)	C2A—C3A—C4A—C5A	−177.8 (2)
O1—C1—C2—C2A	0.8 (3)	C3A—C4A—C5A—C6A	0.6 (4)
C6—C1—C2—C2A	−179.53 (17)	C4A—C5A—C6A—C7A	−0.5 (4)
C1—C2—C3—C4	0.6 (3)	C5A—C6A—C7A—C8A	0.1 (5)
C2A—C2—C3—C4	178.54 (19)	C6A—C7A—C8A—C3A	0.3 (5)
C2—C3—C4—C5	0.7 (3)	C4A—C3A—C8A—C7A	−0.2 (4)
C2—C3—C4—C7	179.9 (2)	C2A—C3A—C8A—C7A	177.4 (2)
C3—C4—C5—C6	−1.0 (3)	C2B—N1B—C1B—C1A^i^	−122.1 (2)
C7—C4—C5—C6	179.8 (2)	C1B—N1B—C2B—C3B	178.98 (18)
C4—C5—C6—C1	0.0 (3)	C1B—N1B—C2B—C6	−3.4 (3)
C4—C5—C6—C2B	179.6 (2)	C5—C6—C2B—N1B	−73.2 (3)
O1—C1—C6—C5	−179.04 (18)	C1—C6—C2B—N1B	106.3 (2)
C2—C1—C6—C5	1.3 (3)	C5—C6—C2B—C3B	104.3 (2)
O1—C1—C6—C2B	1.4 (3)	C1—C6—C2B—C3B	−76.1 (2)
C2—C1—C6—C2B	−178.29 (18)	N1B—C2B—C3B—C8B	158.1 (2)
C2A—N1A—C1A—C1B^i^	126.2 (2)	C6—C2B—C3B—C8B	−19.6 (3)
C1A—N1A—C2A—C2	175.90 (17)	N1B—C2B—C3B—C4B	−20.4 (3)
C1A—N1A—C2A—C3A	−5.3 (3)	C6—C2B—C3B—C4B	161.9 (2)
C3—C2—C2A—N1A	−174.41 (18)	C8B—C3B—C4B—C5B	−0.8 (4)
C1—C2—C2A—N1A	3.5 (3)	C2B—C3B—C4B—C5B	177.8 (2)
C3—C2—C2A—C3A	6.7 (3)	C3B—C4B—C5B—C6B	−1.9 (4)
C1—C2—C2A—C3A	−175.39 (17)	C4B—C5B—C6B—C7B	2.9 (5)
N1A—C2A—C3A—C4A	78.9 (3)	C5B—C6B—C7B—C8B	−1.4 (5)
C2—C2A—C3A—C4A	−102.2 (2)	C4B—C3B—C8B—C7B	2.3 (4)
N1A—C2A—C3A—C8A	−98.6 (3)	C2B—C3B—C8B—C7B	−176.3 (2)
C2—C2A—C3A—C8A	80.2 (3)	C6B—C7B—C8B—C3B	−1.3 (4)

Symmetry codes: (i) −*x*, −*y*+1, −*z*+2; (ii) *x*−*y*+2/3, *x*+1/3, −*z*+7/3; (iii) *y*−1/3, −*x*+*y*+1/3, −*z*+7/3.

### Hydrogen-bond geometry (Å, °)

**Table:**

*D*—H···*A*	*D*—H	H···*A*	*D*···*A*	*D*—H···*A*
O1—H1O···N1A	0.82	1.81	2.532 (2)	146.
